# Long Covid: where we stand and challenges ahead

**DOI:** 10.1038/s41418-022-01052-6

**Published:** 2022-09-07

**Authors:** Alberto Mantovani, Maria Concetta Morrone, Carlo Patrono, M. Gabriella Santoro, Stefano Schiaffino, Giuseppe Remuzzi, Giovanni Bussolati, On behalf of the Covid-19 Commission of the Accademia Nazionale dei Lincei, Pietro Cappuccinelli, Garrett Fitzgerald, Massimo Livi Bacci, Gennaro Melino, Giorgio Parisi, Rino Rappuoli, Giovanni Rezza, Paolo Vineis

**Affiliations:** 1 IRCCS Humanitas Research Hospital, via Manzoni 56, 20089 Rozzano Milan, Italy; 2 Department of Biomedical Sciences, Humanitas University, Via Rita Levi Montalcini 4, 20072 Pieve Emanuele Milan, Italy; 3 William Harvey Research Institute, Queen Mary University, London, EC1M 6BQ UK; 4 Scientific Institute Stella Maris (IRCSS), Pisa, Italy; 5 Department of Translational Research on New Technologies in Medicine and Surgery, University of Pisa, Pisa, Italy; 6 Institute of Pharmacology, Catholic University School of Medicine, and Fondazione Policlinico Universitario “A. Gemelli” Istituto di Ricovero e Cura a Carattere Scientifico (IRCCS), Rome, Italy; 7 Department of Biology, University of Rome Tor Vergata, Rome, Italy; 8 Institute of Translational Pharmacology, CNR, Rome, Italy; 9 Venetian Institute of Molecular Medicine, University of Padua, Padua, Italy; 10 IRCCS Istituto di Ricerche Farmacologiche Mario Negri, Milan, Italy; 11 Accademia Nazionale dei Lincei, Rome, Italy; 12 University of Turin, Turin, Italy; 13 University of Sassari, Sassari, Italy; 14 Institute for Translational Medicine and Therapeutics, University of Pennsylvania, Philadelphia, PA USA; 15 University of Florence, Florence, Italy; 16 School of Medicine, University of Rome “Tor Vergata”, Rome, Italy; 17 Department of Physics, University of Rome ‘La Sapienza’’, Rome, Italy; 18 Head External R&D Division, Glaxo Smith Kline Vaccines, Siena, Italy; 19 Vaccines Research, Imperial College of London, London, UK; 20 Istituto Superiore di Sanità, Rome, Italy; 21 School of Public Health, Imperial College London, London, UK

**Keywords:** Infectious diseases, Immunological disorders

## Abstract

Post-acute sequelae of SARS-CoV-2 (PASC), also known as Post-Covid Syndrome, and colloquially as Long Covid, has been defined as a constellation of signs and symptoms which persist for weeks or months after the initial SARS-CoV-2 infection. PASC affects a wide range of diverse organs and systems, with manifestations involving lungs, brain, the cardiovascular system and other organs such as kidney and the neuromuscular system. The pathogenesis of PASC is complex and multifactorial. Evidence suggests that seeding and persistence of SARS-CoV-2 in different organs, reactivation, and response to unrelated viruses such as EBV, autoimmunity, and uncontrolled inflammation are major drivers of PASC. The relative importance of pathogenetic pathways may differ in different tissue and organ contexts. Evidence suggests that vaccination, in addition to protecting against disease, reduces PASC after breakthrough infection although its actual impact remains to be defined. PASC represents a formidable challenge for health care systems and dissecting pathogenetic mechanisms may pave the way to targeted preventive and therapeutic approaches.

## Facts

- PASC is a frequent legacy of acute SARS-CoV-2 infection, affecting over 10% of patients with different signs and symptoms across a wide range of organs and systems.
- The most frequent manifestations of PASC, in addition to compromised lung functions, include: neurocognitive alterations; alterations of cardiovascular functions and increased risk of acute events; fatigue.
- The SARS-CoV-2 virus seeds and persists in different organs and tissues.
- The pathogenesis of PASC is multifactorial and includes: virus seeding and persistence in different organs; activation and response to unrelated viruses (e.g., EBV); autoimmunity; uncontrolled inflammation.
- Biomarkers of clinical PASC include levels of IgG, cytokines, chemokines, PTX3, and interferons.
- Vaccination reduces PASC after breakthrough infection.

## Open questions

- Occurrence, mechanism, and significance of SARS-CoV-2 persistence in different organs.
- Mechanisms, targets, and significance of autommune reactions.
- Role of other viruses.
- Impact of host genetics and microbiome.
- Actual impact of vaccination in people who get breakthrough infections and its duration.
- Occurrence and severity of PASC after infection with future variants.
- Preventive and therapeutic approaches.

## Introduction

The colloquial terms Long Covid and Post-Covid syndrome (PCS) have been extensively used to identify a wide variety of symptoms occurring for several weeks up to two years following the diagnosis of Covid-19 or symptoms that were consistent with SARS-CoV-2 infection. The syndrome, now formally known as Post-Acute Sequelae of SARS-CoV-2 (PASC), mainly includes neurological and cognitive impairment; fatigue; pain manifestations; cardio-pulmonary symptoms; anosmia-dysgeusia; and headache [1]. The British National Institute for Health and Care Excellence (NICE) defines PASC as “signs and symptoms that develop during or after an infection consistent with Covid-19, continue for more than 12 weeks and are not explained by an alternative diagnosis”. The WHO has crystallized the following clinical case definition of PASC: “it occurs in individuals with a history of probable or confirmed SARS-CoV-2 infection, usually 3 months from the onset of Covid-19 with symptoms and that last for at least 2 months and cannot be explained by an alternative diagnosis” [2].

**Fig. 1:**
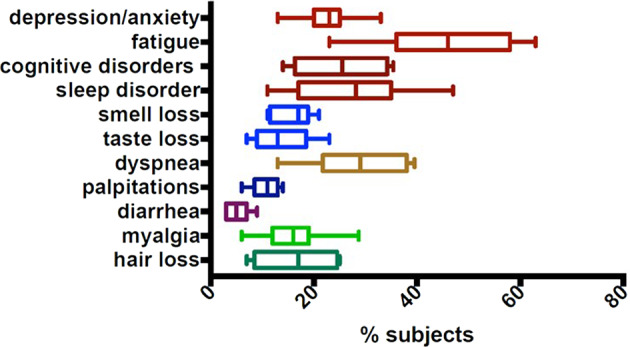
The frequency of the most common symptoms four week or more after the acute Covid-19 infection. Data presented in meta-analyses, selected on the basis of homogeneous reporting criteria [10–20].

Women and men were affected differently from the Covid-19 pandemic. Women did show less severe complications in the short-term while suffering from worse long-term ones such as depression, impaired physical activity impacting also on lifestyle habits, increasing the cardio-vascular risk [3, 4]. The impact of COVID-related long-time imbalances affecting the general population should not be under-estimated [5]. In fact, the incidence of people reporting COVID-related symptoms varies extensively, being related to sex, age, and severity of symptoms during the acute phase. In UK, epidemiological analyses conducted by February 2021 estimated that from 1 up to 2 million people reported at least one COVID-related symptom which lasted for 12 or more weeks [6]. According to a metanalysis conducted in UK, the main symptoms still present 1 year after the acute disease included cognitive and mental health disorders, such as depression, anxiety, memory loss, concentration difficulties and insomnia, fatigue, dyspnea, muscle, and joint pain [7]. A higher risk of diabetes has also been observed [8]. Female sex and severe COVID-19 disease were associated with higher risk of experiencing symptoms of PASC [8]. In a large population study in Southern Germany (EPILOC cohort, age 18–65), the three most frequent clusters of symptoms were fatigue, neurocognitive, and chest/cardiorespiratory, with at least moderate impairment (>20%) of general health and working capacity in 26% of the subjects (age and sex standardized), including young and middle-aged subjects [9]. Numerous reports have dealt with the frequency of symptoms related to SARS-CoV-2 and present several weeks or months following the acute phase. Most reports are limited in number and meta-analyses can provide more extensive and reliable values. Frequencies collected from 11 meta-analyses reporting homogeneous information [10–20] allow to present reliable data (Fig. 1). Information on the presence of Covid 19-related symptoms over 12 months from the acute infection are limited [8, 10] but confirm the possibly of persistence of symptoms (especially fatigue and cognitive disorders) for long time.

Symptoms, related pathological findings, pathogenetic data, and prognostic prospects are different in different organs, which implies separate analytical reports of patterns involving lung, cardio-vascular system, neuro-muscolar system, and brain. Other organs can also be affected. Besides the already mentioned higher frequency of diabetes, kidney imbalances have been reported. Adult patients who survived Covid-19 beyond the first 30 days of infection exhibited increased risk (and burden) of acute kidney insufficiency, eGFR (estimated Glomerular Filtration Rate) decline, End Stage Kidney Disease, and major adverse kidney events [21]. The risk (and burdens) of kidney outcomes increased according to the severity of the acute infection.

Here we will review the current understanding of the main manifestations and organ involvement by PASC. Underlying cellular and molecular mechanisms will be discussed with emphasis on the contribution of viral persistence and immune responses.

## Organs and systems involved

### Lung

Persistent dyspnea, frequently associated with fatigue, chest pain, and cough affect ~20% of patients 3 months after the acute SARS-CoV-2 infection [22]. Lung involvement by PASC is generally related to the disease severity, but there is no strict relationship between dyspnea and the degree of initial disease [23]. In most cases, dyspnea progressively improves over time even if a subgroup of patients experiences persistent dyspnea up to 1 year after Covid-19. Interestingly, among Survivors of Covid-19 experiencing long-term symptoms, increased breathlessness, and reduced quality of life were observed in young, previously healthy working age adults and frequently younger females [24].

After hospital discharge, fatigue, dyspnea, chest pain, and cough are the most prevalent respiratory symptoms found in 52%, 37%, 16%, and 14% of patients between 3 weeks and 3 months. The pathogenesis of persistent COVID-related symptoms is likely multi-factorial, but evidence indicates that pulmonary endotheliopathy and pro-thrombotic changes, as well as inflammatory cytokine production could be involved [22, 25, 26]. Abnormalities in alveolar diffusion capacity, revealed by diffusing lung capacity for carbon monoxide (DPCO) tests persist for long periods and are likely related to interstitial pneumonia, which might evolve into pulmonary fibrosis. A long-term evolution towards pulmonary fibrosis is a possible occurrence, strictly related to the severity of pulmonary inflammation during the acute phase and affecting which higher frequency patients which required mechanical ventilation. Radiological data are important in the management of Covid-19 patients and in the follow-up after the acute phase. Interstital fibrosis leads to abnormalities at high-resolution CT-scans: reticulations and traction bronchiectasis can reveal the severity of the process, months after the acute infection. McGroder and Coworkers [27] observed that such radiological abnormalities were related with cough and pulmonary function degradation. Furthermore, these Authors reported that fibrotic-like radiological abnormalities correlated with shorter blood leukocyte length. The respiratory outcome of patients who required hospitalization during the acute phase tends to improve over time, as assessed by radiological exams and pulmonary function tests such as DLCO (diffuse capacity of the lungs for carbon monoxide) but in a fraction of patients changes persisted up to 1 year [28].

Diffuse alveolar damage in the proliferative phase and collagen deposition were observed in a series of autopsies performed on patient died over 65 days after infection. Pulmonary tissue damage can be amplified by concomitant bacterial superinfection, aspergillosis, thromboembolism, and hemorrhage [29].

A study on immuno-fibrotic drivers of impaired lung function in PASC reports that circulating factors associated with acute neutrophil activation, fibrosis signaling, and alveolar epithelial repair remain elevated in survivors of acute Covid-19 infection and may predict the impairment of pulmonary function [30].

A meta-analysis including a total of 4478 Covid-19 patients from 16 cohort studies reports that fatigue or weakness (47%) were the most prevalent physical effects of post-acute Covid-19 syndrome. In recovering patients, defective lung functionality as revealed for instance by diffusion capacity for carbon Monoxide (DLCO < 80%) persisted for long time. Decreased lung function and joint pain were more frequently observed in patients with severe disease [16].

### Cardio-vascular system

Myocardial injury associated or not with the multisystemic inflammatory syndrome [31] occurs frequently in patients with acute Covid‐19 infection (as revealed also by high serum Troponin levels) and is associated with increased mortality during hospitalization. In the general population, an incidence of Covid-19-associated myocarditis of ~150 cases per 100,000 was observed [32]. In patients who survive, the incremental mortality at 6 months and 1 year was seen to be low [33]. Some evidence indicates that males between 12 and 17 years of age most likely developed myocarditis within 3 months of SARS-CoV-2 infection [34].

An accurate statistical analysis estimated the risks and 12-month burdens of pre-specified cardiovascular outcomes confirming that they are substantial and span several cardiovascular disease categories (ischemic and non-ischemic heart disease, dysrhythmias, and others). Symptoms may include chest pain, shortness of breath, fatigue, and autonomic manifestations such as postural orthostatic tachycardia which are common and associated with significant disability, heightened anxiety, and public awareness [35–37]. The risks and burdens of cardiovascular disease were evident even among patients who did not necessitate hospitalization for acute Covid-19 disease [38]. Patients with PASC frequently experience Inappropriate Sinus Tachycardia (IST), possibly sustained by a cardiac autonomic nervous system imbalance with decreased parasympathetic activity [39]. Most cardiac abnormalities were seen to alleviate with time, but some of them, especially diastolic dysfunction, may persist, raising the presumption of a chronic alteration [40].

The pathogenesis for post-acute cardiac damage is still not fully elucidated. Possibly, a chronic inflammatory response evoked by persistent viral reservoirs in the heart after acute infection might be the explanation, with underlying mechanisms suggested for post-acute Covid disease affecting other organs (see below). Moreover, another putative mechanism for delayed damage is an autoimmune response to cardiac antigens through molecular mimicry, and some evidence has been presented in favor of this hypothesis [41].

### Neuromuscular system

Muscle weakness, fatigue, and exercise intolerance are among the most frequent symptoms of PASC. Myalgia is also observed in several patients and the symptoms may persist for several weeks or months [42]. This condition, which is more frequent in patients who were hospitalized for Covid-19, but is also seen in non-hospitalized patients, is similar to the chronic fatigue syndrome (CFS), also called myalgic encephalomyelitis (ME) or ME/CFS that may occur following different viral infections, thus also referred to as post-viral fatigue syndrome (PVFS). The pathogenesis of all these conditions is unclear. To dissect the causes of muscle fatigue in PASC, it is useful to consider the pathogenesis of neuromuscular symptoms also during the acute phase of severe Covid-19.

#### Muscle wasting in Covid-19 patients admitted to ICU

Intensive-care patients with severe Covid-19 show dramatic muscle wasting and weakness, a condition related to the Critical Illness Myopathy due to immobilization and mechanical ventilation seen in many patients admitted to ICU, independently of the cause of the disease [43]. This condition is followed, for those who survive, by sustained physical disability and requires a long rehabilitation process. Both myogenic mechanisms, with loss of myosin from the muscle fibers, and neurogenic factors, with slowing of nerve conduction velocities and axonal degeneration, may contribute to the Critical Illness Myopathy seen in patients with severe Covid-19 [42]. In addition, other factors, including systemic inflammation with increased cytokine levels (cytokine storm), hypoxemia, which is present in all patients with severe disease, malnutrition due to loss of appetite, loss of smell, and alteration in taste, likely contribute to promote muscle wasting.

#### Viral infection of skeletal muscles

It is not clear whether viral infection of muscles is involved in muscle changes during and after Covid-19. Evidence for myositis has been reported in deceased patients with Covid-19. However, detection of viral load was low or negative in most skeletal muscles, and probably attributable to circulating viral RNA rather than direct infection of muscle cells [44].

#### Peripheral neuropathy during or after SARS-CoV-2 infection

Several Covid-19 patients show symptoms of peripheral neuropathy, such as painful paresthesia (numbness and tingling) either during or after SARS-CoV-2 infection [45]. In some of these patients, a diagnosis of small fiber neuropathy was supported by skin biopsy, and autonomic dysfunction was demonstrated by autonomic function testing. Combined involvement of motor and sensory nerves was seen only in occasional patients, for example patients showing bifacial weakness and paresthesia [46]. These cases are consistent with conditions related to various forms of Guillain-Barré syndrome (GBS), probably caused by autoimmunity, thus different from other sensory disfunctions seen in Covid-19, such as anosmia and dysgeusia, which seem to reflect a direct viral infiltration of the nervous system.

### Nervous system

It is now clear that many brain functions are affected for a long time after Covid-19 infection, in patients both with severe and mild symptomatology [47, 48]. PASC includes cognitive, neurological and psychiatric diseases, and distressing symptoms such as memory loss, fatigue, anosmia, and dysgeusia. The peculiar sensory deficits, anosmia/dysgeusia, that characterized the early symptoms of Covid-19 were manifested in more than 40% of Covid-19 patients infected with Delta or previous variants. It affected patients of all ages and the impairment lasted on average for 2–3 months after the end of the infection. However, even in young adults with no severe Covid, loss of taste and/or smell (about 28% of prevalence) were present at 6 months post infection. These sensory deficits are amongst the brain function deficits with a faster recovery in PASC [49, 50].

Cognitive dysfunction in PASC is very broad, affecting attention, executive function, problem solving, and decision making. The most prevalent dysfunction concerns memory, affecting up to 73% (in an interview study on 2739 patients), inducing both short-term and long-term memory loss [50]. The time course of the loss and of the possible recovery of the many affected brain functions are variable: cognitive dysfunction increased over the first three months post infection, then decreased slightly in the following 7 months. The probability of experiencing memory symptoms increased over the first few months, with 56% reporting memory symptoms at month 4 and 50% at month 7. While age is an important factor in cognitive and memory disfunction, it is worrying that non-hospitalized, young people (16–30 years old) suffer potentially severe symptoms, such as concentration and memory problems, half a year after infection [47, 50].

The study of the anatomical or functional imaging of brain alterations in PASC shows consistent changes in many brain areas, including the somatosensory cortex, rectal/orbital gyrus (including the olfactory system), temporal lobe (including the amygdala, piriform cortex, and the hippocampus), hypothalamus/thalamus, brainstem, and cerebellum [51]. 18F-FDG brain PET studies in Covid-19 patients have shown prominent hypometabolism in many of the above areas. However, during the PASC phase, a reversibility of the decreased neocortical glucose metabolism is evident, which importantly is associated with an improvement in cognitive function. Interestingly, the spatial covariance pattern of the hypometabolism correlates with the cognitive impairment [52].

The preliminary evidence of brain alterations has been corroborated by a larger study that could compare in the single patient (55–75 years of age) brain anatomy before and about 5 months after the Covid-19 infection [53]. Again, this study recruited patients in 2020 and early 2021, and hence does not include infections with omicron variants. Both gray and white matter of many brain areas change. The changes are subtle but are consistent across individuals and highly significant. Gray matter, evaluated by cortical thickness, is reduced in many regions of the orbito/frontal cortex and limbic system that include olfactory cortex, piriform cortex, amygdala, parahippocampal and hippocampal cortex, and insula. The changes are consistent with the white matter alterations, measured with mean diffusivity, in regions functionally connected with the piriform cortex, olfactory tubercle, and anterior olfactory nucleus. These altered structures participate in the perception of taste, smell, emotion, memory, and spatial navigation, functions that are strongly compromised during PASC.

The correspondence between the major cognitive and neurological dysfunction in PASC and the neuronal substrate that mediates these functions suggest that the observed symptoms result from insults, although small, to the brain in consequence of the infection. The mechanism that generates the insults is still to be defined (see below).

A pronounced loss of gray matter was also observed in crus II, part of the cognitive, and olfactory-related lobule VII of the cerebellum. Interestingly, the amount of gray matter loss correlated well with the patient individual performance in a spatial attention task widely used as a neuropsychological test. Again this demonstrates a causal link between brain alterations and behavioral deficits. Despite these highly localized deficits, there is also an increase in Cerebro Spinal Fluid (CSF) volume and decrease of whole brain volume respect to the controls, suggesting an additional diffuse loss of gray matter. The anatomical deficits increase with age between 60 and 75 and are likely to be modest in the age group of 55. This reinforces neuropsychological data that showed Covid-19 as a risk factor to develop dementia, neurodegenerative diseases and mild cognitive impairments even in 50-year-old adults [54].

### Metabolic dysfunctions and diabetes

Metabolic dysfunctions, such as obesity and insulin resistance, and metabolic diseases, such as diabetes, were recognized as predisposing risk factors for severe acute Covid-19 since the early stages of the pandemic. Now emerging evidence supports the notion that these conditions also predispose to Long Covid (PASC). For example, lipid metabolism disorders and obesity were found to be age-independent risk factors for the development of PASC, as shown in a retrospective study involving more than 50.000 patients with a confirmed diagnosis of Covid-19 treated by general practitioners in Germany [55].

Type 2 diabetes is a well-established PASC-anticipating risk factor [49] and several reports now support the notion that the incidence of diabetes is increased after Covid-19 [56]. Abnormalities in glycometabolic control, insulin resistance, and beta cell function were detected in patients with Covid-19 without any pre-existing history or diagnosis of diabetes and persist even after recovery [57].

The study by Xie and Al-Aly [7] stands out for its large sample size. A cohort of more than 180,000 participants who had a positive COVID-19 test were followed up for about 1 year. Compared to a non-infected contemporary and a historical control group (both > 4 M subjects) cohort members were observed to have an increased risk of incident diabetes [7]. The risk was found to increase according to the severity of disease during the acute phase of the infection, Comparing three groups of patients (non-hospitalized, hospitalized, and admitted to intensive care), the risk was found to be related to disease severity but also present in the non-hospitalized group. The excess burden of diabetes among non-hospitalized individuals (8.3 per 1000 people at 12 months) points to the magnitude of the problems that health systems might face, considering the hundreds of millions of people infected globally. Given the importance of this major risk of Long Covid, it will be important to support the conclusion of these reports with prospective epidemiological studies [58]. An immediate implication of the studies is the necessity of screening for hyperglycemia not only during the acute phase of Covid-19 but also during the follow-up.

## Pathogenesis and biomarkers

**Fig. 2:**
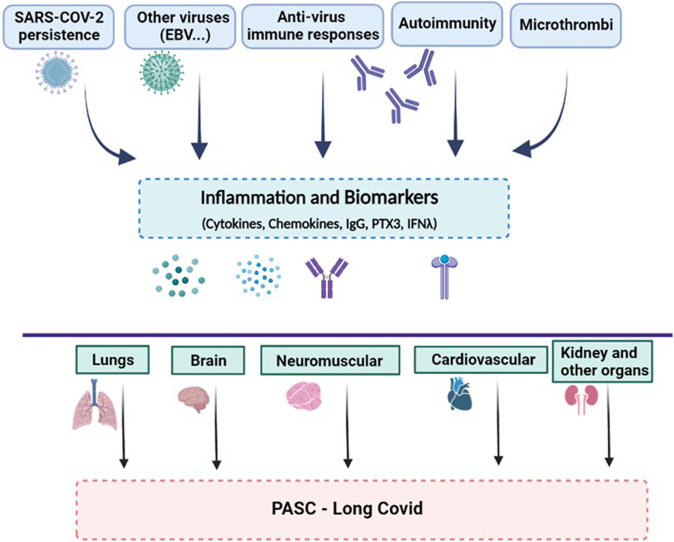
A schematic representation of the pathogenic mechanisms and main targets of PASC. The drivers, effector molecules, biomarkers, and affected organs are presented in a schematic form, as discussed in the text. The inset suggesting that the host genetics and microbiome may affect the development of Long Covid is based on current evidence on determinants of severe Covid-19. Type 2 diabetes has been shown to increase the risk of developing PASC.

Understanding fundamental mechanisms underlying the pathogenesis of Long Covid is in its infancy and represents a major challenge. Candidate mechanisms of pathogenesis can be classified along five major lines: persistence of SARS-CoV-2; reactivation of other viruses, in particular Epstein-Barr virus (EBV); autoimmunity triggered by the virus; persistent tissue damage and immunity-triggered inflammation [59, 60]; formation of microthrombi in the vascular bed of different tissues [61] (Table 1; Fig. 2).

**Table 1:** Mechanisms of pathogenesis of PASC.

Mechanisms	Details	Selected refs.
Persistence of SARS-CoV-2 and/or fragments	High RNAmia at the time of diagnosis is a risk factor	[49]
	SARS-CoV-2 presence in different organs	[62, 85]
	Viral persistence in the GI tract	[63]
Activation of other viruses	Circulating EBV	[49]
	CMV reactive T cells	[49]
Innate immunity, inflammation	Myeloid cell activation, cytokines (IFN, IL-6), PTX3	[73, 74]
	Glial cell disregulation; TNF, IL-6, CCL11; brain fog	[83]
Adaptive immunity	Antibodies (IgG/IgM signature, T cell activation)	[49, 72]
Autoimmunity	Autoantibodies, autoreactive T cells	[70, 71]
Microclots	Endothelial cell and virus triggered microthrombi	[61]

Persistence of the virus and viral fragments has been proposed to represent a driver sustaining long-term sequelae of PASC (see below) [62]. Gastrointestinal (GI) viral shedding has been associated in some patients with persistent disease following the acute phase of the disease [63]. Consistent with the view of an important role of virus persistence are preliminary observations of vaccination of Long Covid patients being associated with resolution [64, 65]. PASC occurs in the aftermath of a complex interplay between the virus and the host immune system (see for review [60, 66]). Intriguingly, a mechanism of subversion of immunity by SARS-CoV-2 includes syncityum-mediated lymphocyte elimination [67, 68].

Consistent with an important role of the virus itself, SARS-CoV-2 RNAmia was recently identified as a risk factor for PASC at the time of initial diagnosis [49]. In this longitudinal cohort of 209 patients investigated using a multi-omic approach, additional risk factors included diabetes, circulating EBV, and auto-antibodies. The EBV data suggest that viral reactivation may contribute to the pathogenesis.

Immune activation and autoimmunity have long been associated with PASC [69]. Indeed, autoantibodies have been shown to contribute to severe Covid-19 disease [70]. Activation of autoreactive T cells has been observed in infection settings including Covid-19 [71]. In a recent study, GI-PASC was found to correlate with newly expanded cytotoxic CD8+ and CD4+ T-cell populations. These new populations include SARS-CoV-2 reactive clones and their activation occurred during convalescence from acute disease. Concomitantly, non-specific activation of CMV-specific T cells was observed in subjects with GI-PASC [49].

A limited number of prospective studies with validation cohorts have been conducted to investigate pathogenesis and prediction of evolution to PASC. In a study involving 215 subjects and a 395 individuals validation cohort [72], an antibody signature (IgM and IgG3) together with a set of clinical variables was able to predict PASC. A study conducted on 147 patients in addition to normal subjects, included controls who had been infected with prevalent coronaviruses other than SARS-CoV-2 [73]. Eight months following mild-to-moderate SARS-CoV-2 infection profound perturbations were found in Covid-19 patients. Myeloid cells showed an activated phenotype and alterations of naive T cells were observed. A combination of analyses was associated with PASC with a 78–81% accuracy. This set of biomarkers included cytokines (IFN-β, IFN-γ, IFN-λ, and IL-6) and the fluid phase pattern recognition molecule PTX3 [74].

Covid-19 has been associated with microvascular thrombosis [75–80] and microthrombi have been suggested to play a role in PASC [61]. Different mechanisms may contribute to formation of microclots. Endothelial cell activation and activation of the lectin pathway can facilitate thrombus formation [61, 75–81]. Fibrinogen in platelet-poor plasma of PASC patients has been shown to clot in an anomalous “amyloid” form of fibrin resistant to fibrinolisis. A propensity to develop microthrombi in PASC has obvious implications for the pathogenesis of cardiovascular problems discussed above. Intriguingly, it has been suggested that the SARS-CoV-2 proteome includes amyloidogeneic peptides which may contribute to neurological symptoms [82].

Brain fog is a prominent feature of PASC and a recent study identified a cytokine/chemokine cascade as a driver of its pathogenesis [83]. In mice, mild respiratory Covid triggered microglial reactivity with loss of neurogenesis and of myelinated axons. Neuroinflammation was sustained by cytokines (TNF and IL-6) and a chemokine (CCL11). In agreement with these data in mice, humans with lasting cognitive symptoms after Covid-19 showed elevated levels of CCL11.

Thus, the pathogenesis of PASC is complex, at the interception between virus persistence, activation of, and response to, endogenous viruses (EBV and possibly others), activation of antiviral and autoimmune responses, sustained inflammation. Given the diversity and pleiomorphic nature of PASC manifestations, it is tempting to speculate that the relative importance of different pathogenic components may vary depending on the spectrum of organs involved.

## Mechanisms of pathogenesis: virus persistence

**Fig. 3:**
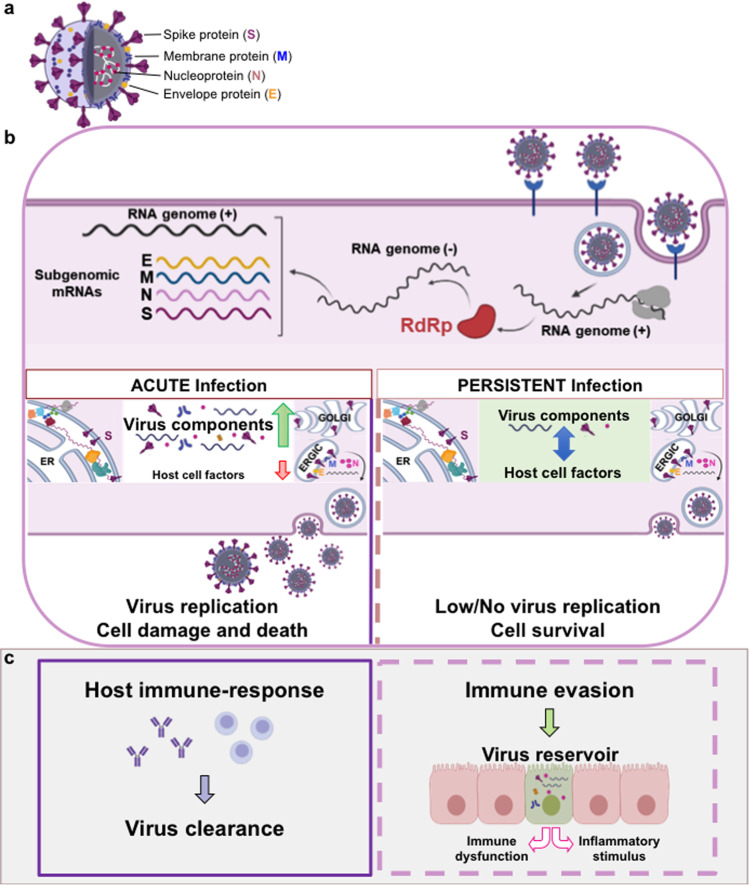
Schematic representation of SARS-CoV-2 virus infection and its role in PASC. **a** The SARS-CoV-2 virus lipid bilayer comprising the spike protein (S, violet), the membrane protein (M, blue) and the envelope protein (E, orange), and the viral RNA (white) associated with the nucleocapsid protein (N, pink) are shown. **b** Different steps of SARS-CoV-2 replication cycle are illustrated in the cartoon, including binding to the ACE2 receptor (blue), virus entry, viral RNA replication, sub-genomic RNA transcription and translation, virus assembly, and exit from the host cell. RdRp, RNA-dependent RNA polymerase. ER endoplasmic reticulum, ERGIC ER-Golgi intermediate compartment. During acute infection (right), the virus hijacks the host cell transcriptional/translational machinery to make large amounts of viral proteins and RNA (green arrow), while shutting down cellular protein synthesis (red arrow), resulting in infectious virus progeny production, and host cell damage and death. The host immune-response eventually leads to virus clearance (gray box, **c**). The mechanisms at the basis of virus persistence in the host cell are currently unknown. In the hypothetical model of persistent infection (left) concurrence of molecular and immunological events may allow a metastable equilibrium between SARS-CoV-2 and the host cell (blue arrow), where a virus-directed transcriptional program enables a long-lasting virus-host interaction and cell survival. Evasion of the host immune response may allow the establishment of virus reservoirs (gray box, **c**). In persistently infected cells viral RNA and/or selected viral proteins might act as constant stimuli causing chronic immune system dysregulation and inflammation (**c**, left panel).

A growing number of studies provide evidence that in some PASC patients, SARS-CoV-2 is capable of persisting in several tissue reservoirs after acute infection. In addition to the respiratory tract, SARS-CoV-2 viral proteins and/or RNA have in fact been detected throughout the cardiac and renal systems, GI tract, muscles as well as in the brain and lymph nodes months after infection (reviewed in [84]) (Fig. 3).

Recently, in one of the most comprehensive analyses to date of SARS-CoV-2 persistence across the body and brain in a diverse autopsy cohort collected in the United States, the authors report that, whereas the most common location in which SARS-CoV-2 RNA tends to linger is the respiratory tract, in more than 50% of the cases the virus was detected also in extrapulmonary tissue, including in the myocardium, lymph nodes and in all sampled areas of the brain, except the dura mater [85]. The data also indicate that SARS-CoV-2 can replicate within different tissues for over 3 months after infection. In some individuals, viral RNA could be detected in multiple compartments for up to 230 days after primary infection [85]. The authors suggest that the persistence of viral genomic and subgenomic RNA may represent infection with defective virus, which has been described in persistent infection with other viruses, including the measles virus.

In addition to autopsy findings, persistence of SARS-CoV-2 RNA was detected in intestinal enterocytes of 5 out of 14 intestinal biopsies obtained from asymptomatic individuals at 4 months after the onset of Covid-19 [86]. Interestingly, a recent study also revealed the presence of virus transcripts and of SARS-CoV-2–infected cells in the olfactory mucosa of patients with long-term persistence of Covid-19–associated anosmia who were negative to nasopharyngeal swab SARS-CoV-2 RNA tests [87].

Persistence of SARS-CoV-2 in some Covid-19 patients is not unexpected. Several studies have shown that coronaviruses are capable of establishing persistent infections in vitro as well as in vivo. Starting from the initial studies on the beta-coronavirus MHV (murine hepatitis virus) that was extensively investigated for its ability to cause persistent infection in the central nervous system also in primates, in some cases associated with demyelination [88, 89], several studies have shown that persistent infection of FCoV (feline coronavirus) can often occur in cats [90]. Regarding human coronaviruses (HCoV), the ability of establishing persistent infection in cell cultures has been demonstrated for the seasonal coronaviruses HCoV-OC43 and HCoV-229E [91, 92], as well as for the SARS-CoV-2 phylogenetically related SARS-CoV and MERS-CoV [93, 94]. In the case of these two highly pathogenic coronaviruses, it should be noted that a subset of individuals who survived SARS or MERS were reported to experience, in addition to persistent impairment of pulmonary function, protracted neuropsychiatric symptoms, sleep abnormalities, fatigue, myalgias and functional disabilities reminiscent of Long Covid (reviewed in [84]).

In the case of SARS-CoV-2, it has been recently shown that the virus can establish a long-term, non-productive persistent infection in different types of cells [95, 96].

The molecular mechanisms governing the establishment of RNA virus persistent infections have attracted considerable attention, but remain elusive. In the case of SARS-CoV-2, during acute infection the virus hijacks the host cell transcriptional/translational machinery to make large amounts of viral proteins and RNA, while shutting down host messenger RNA translation [97, 98], resulting in infectious virus progeny production and cell death; during persistent infection it is hypothesized that concurrence of molecular and immunological events is required to allow the virus to direct a transcriptional program enabling a long-lasting virus-host interaction, by regulating its replication without killing the host cell and by evading the immune response (Fig. 3). Establishment of SARS-CoV-2 persistent infection has been associated with immunosuppression [99, 100], reduced expression of ribosomal proteins [100] and possible integration of selected SARS-CoV-2 sequences into the genome of infected cells [101].

Another intriguing hypothesis to be considered is that, due to the high cell–cell fusion activity of its spike protein [102, 103], the SARS-CoV-2 virion or some of the virus components may spread through cell–cell contact. This insidious strategy, which is adopted by other RNA viruses, including the respiratory syncytial virus, the measles virus [104] and the human immunodeficiency virus [105], allows the pathogen to spread in a particle-independent way, promoting immune evasion [106]. Cell-to-cell transmission of SARS-CoV-2 has been recently demonstrated in human cells [107].

Contribution of SARS-CoV-2 persistence to PASC pathogenesis is not currently understood, but it could be hypothesized that viral RNA and/or selected viral proteins might act as constant stimuli that maintain an inflammatory condition contributing to pathogenesis until viral clearance is achieved (see above). This possibility is supported by reports of improved clinical symptoms after administration of anti-SARS-CoV-2 vaccines in PASC patients [65].

## Protection by vaccination

A preliminary patient-led observational study has suggested that PASC symptoms might be diminished through vaccination [108]. Among 900 people affected by Long Covid, 56.7% of the vaccinated saw an overall improvement, 18.7% a deterioration, and 24.6% were unchanged post-vaccination. A different survey (Covid symptom app study) [109] showed that the odds of experiencing symptoms more than 28 days post-vaccination, were halved by two vaccinations (*n* = 906). It has been suggested that an increased viral clearance and a muted chronic inflammatory response could explain the reduction of symptoms after vaccination [110]. Early evidence was obtained in Israel that childhood vaccination against Covid-19 protects against both, the direct acute and the long-term effects of Covid-19 disease [111].

Three recent studies have investigated the impact of vaccination on PASC following breakthrough infection (BTI). In a large study conducted on the US Department of Veterans Affairs database it was observed that vaccination with a single dose of the Ad26.CoV2.S or two doses of a mRNA vaccine conferred only limited, but significant, protection against Long Covid after BTI [112]. Limitations of this study include the time window of observation (January through October 2021), the low number of females (<10%), the suboptimal vaccine schedule. A longitudinal study involving a carefully controlled hospital personnel cohort conducted in Italy covering the omicron sustained wave in spring 2022 indicated strong protection against PASC after BTI by vaccination with mRNA vaccines [113]. The observation time included the spring 2022 wave and protection was dependent on the number of jabs, requiring two or three shots. Protection by vaccination against PASC after BTI was also observed in a survey on Long Covid [114] conducted in Israel.

Assessment of protection against PASC after BTI poses methodological challenges with limitations which are inherent to longitudinal versus case-control studies, usage of different vaccines or number of jabs, representation of different prevailing virus variants. However, in spite of these limitations, available information obtained using different approaches strongly suggests that full vaccination with mRNA vaccines confers protection against the development of PASC after BTI. The duration of protection and its significance to future variants remains to be defined.

## Concluding remarks and perspective

Progress has been made in defining key cardinal aspects of PASC (neurocognitive, cardiorespiratory, fatigue, etc.) and its prevalence, but important aspects remain undefined. These include the actual boundaries of the PASC symptom constellation, its similarity and peculiarities in relation to other viral diseases, its actual frequency and relevance in the pediatric population.

Some of the symptoms and imbalances characteristic of PASC tend to last up to months, but are ultimately going to disappear, although in a minority of patients, anosmia, brain “fog”, DPCO, and dyspnea can persist after one year even among young and middle-aged adults after mild acute SARS-CoV-2 infection and impact on general health and working capacity [115–117]. Females showed significantly more neurocognitive symptoms than males. It has been observed that among patients symptomatic after 2 months, 85% still reported symptoms one year after their symptom onset, while evolution of symptoms showed a decreasing prevalence over time for 27/53 symptoms (e.g., loss of taste/smell); a stable prevalence over time for 18/53 symptoms (e.g., dyspnea), and an increasing prevalence over time for 8/53 symptoms (e.g., paresthesia) [118]. Of major concern are the reported increase in incidence, following Covid-19 infection, of Diabetes and cerebrovascular events, notably acute ischemic strokes. In addition, Covid-19 is a risk factor for deep vein thrombosis, pulmonary embolism, and bleeding [119] and coagulopathies (dysfunctions of the blood coagulation system), possibly related to fibrin amyloid microclots [61], that persist long after the initial infection. Alterations (reduction in thickness) of the brain cortex as a sequel of Covid-19 infection was observed in specific areas, mainly related to olfact sensibility, but it is not known if such derangements are going to persist in time. Recent data (May 2022) from Wuhan indicate that Covid-19 survivors still had more prevalent symptoms and more problems in pain or discomfort, as well as anxiety or depression, at 2 years than did controls [120].

Most of the reported observations on the sequels to Covid-19 infections are related to early variants of the virus: we do not know and only time will tell if the now prevailing omicron variants induce similar effects [9, 120]. It is tempting to speculate that the lower intrinsic pathogenicity of omicron and the dramatic impact on disease severity of vaccination will translate in lower risk of PASC at the individual level. Recent results suggest that early omicron variants are associated with approximately a 50% reduction in the risk of developing PASC compared to delta [121]. However, given the increase in transmission of omicron variants, including children, the potential PASC disease burden at the population/society level should not be underestimated and deserves careful assessment.

Current understanding of pathogenesis is in its infancy. Evidence suggests that persistence of Covid-19, reactivation of other viruses, autoimmunity, and uncontrolled inflammation are major determinants of PASC. Given the diversity of organ involvement and manifestations, it is tempting to speculate that the relative importance of pathogenic mechanisms may vary in different tissue and organ contexts. A better understanding of the PASC disease spectrum and underlying mechanisms may pave the way to better prevention and therapeutic strategies. It is reasonable to assume that prevention via vaccination and early treatment of the acute phase of Covid-19 represent invaluable assets to address the challenge of PASC at the level of individuals and society.
